# Supplementary material for: Effect of yeast and essential oil-enriched diets on critical determinants of health and immune function in Africanized Apis mellifera
Source: PeerJ. 2021 Oct 15;9:e12164. doi: 10.7717/peerj.12164 (PMC8522645; doi:10.7717/peerj.12164)
Supplement: Supplemental Information 1 — Nucleotide sequences of Starmerella bombicola (437bp, 358, B), Starmerella etchellsii (393bp, 386, C), Starmerella bombicola 2 (388bp, 413, D), Zygosaccharomyces mellis (542bp, 443, E), and Saccharomyces cerevisiae (519bp, Sac2 brewing strain, F). [file peerj-09-12164-s001.docx]

**Appendix**

Nucleotide sequences of the studied strains in Canche-Colli, et al.

Strain CICY-RN-358: *Starmerella bombicola* (437bp, 358, B) D1/D2 LSU rDNA gene partial sequence. Strain isolated from stored honey in the nest of *Scaptotrigona pectoralis* (Apidae). Accession number of GenBank: MW267941

CTTAGGTCCTCGCGCCCGAGTGGGAGTATGCACTGTAGCTATAACACTCCGAAGAGCCACATTCTACAGCTTTTATCCTCCCCTCAGACACGGCTCTACGTGGTTAGCGGCCTACCCTTCCATTTCAACAATTTCACGTACTTTTTCACTCTCTTTTCAAAGTTCTTTTCATCTTTCCTTCACAGTACTTGTTCGCTATCGGTCTCTCGCAGATATTTAGCTTTAGATGGAGCATACCACCCATTTGAGCTGCATTCCCAAACAACTCGACTCCACGCCAAGATTCTACAATGAGGTCAGTACCGTACGGGGCTATCACCCTCCTTGGCGCTCCTTTCCAGAAGACTTAGGTACCGGTTCTCAGAATCAAGGCTTCAGAATACAATGCCCCGAGAGGCTTTCAAATCTGAGCTTTTGCCTGTTCACTCGCCGTTACT

Strain CICY-RN-386: *Starmerella etchellsii* (393bp, 386, C) D1/D2 LSU rDNA gene partial sequence. Strain isolated from food deposited in brood cell in the nest of *Scaptotrigona pectoralis* (Apidae). Accession number of GenBank: MW267942

CTTCAGCTTTTGTCTTCCCCCCAAACACGGCTCTACATTGTTAGCGGCATATCCTTCCATTTCAACAATTTCACGTACTTTTTCACTCTCTTTTCAAAGTTCTTTTCATCTTTCCTTCACAGTACTTGTTCGCTATCGGTCTCTCACAGATATTTAGCTTTAGATGGAGCATACCACCCTTTTGAGCTGCATTCCCAAACAACTCGACTCCACGTCAGAATCCTACAATGAGTGAGGTGTCGTACGGGGCTATCACCCTCCATGGCGCTCCTTTCCAGAAGACTTAAACACCGATACTCAGGAAACTGACTTCAGAATACAATGCCAAAAAGGCTTTCAAATCTGAGCTCTTGCCTGTTCACTCGCCGTTACTAGGGCAATCCCTGTTGGTTT

Strain CICY-RN-413: *Starmerella bombicola* 2 (388bp, 413, D) D1/D2 LSU rDNA gene partial sequence. Strain isolated from stored pollen in the nest of *Scaptotrigona pectoralis* (Apidae). Accession number of GenBank: MW267943

TCAGATTTGAAAGCCTCTCGGGGCATTGTATTCTGAAGCCTTGATTCTGAGAACCGGTACCTAAGTCTTCTGGAAAGGAGCGCCAAGGAGGGTGATAGCCCCGTACGGTACTGACCTCATTGTAGAATCTTGGCGTGGAGTCGAGTTGTTTGGGAATGCAGCTCAAATGGGTGGTATGCTCCATCTAAAGCTAAATATCTGCGAGAGACCGATAGCGAACAAGTACTGTGAAGGAAAGATGAAAAGAACTTTGAAAAGAGAGTGAAAAAGTACGTGAAATTGTTGAAATGGAAGGGTAGGCCGCTAACCACGTAGAGCCGTGTCTGAGGGGAGGATAAAAGCTGTAGAATGTGGCTCTTCGGAGTGTTATAGCTACAGTGCATACTCC

Strain CICY-RN-443: *Zygosaccharomyces mellis* (542bp, 443, E) D1/D2 LSU rDNA gene partial sequence. Strain isolated from stored honey in the nest of *Scaptotrigona pectoralis* (Apidae). Accession number of GenBank: MW267944

TTAGTAACGGCGAGTGAAGCGGCAAGAGCTCAAATTTGAAATCTGGTACCATTCGGTGCCCGAGTTGTAATTTGGAGAGAGCGATTCTGGGGCTGGCGCTTGCCTATGTTCCTTGGAACAGGACGTCATAGAGGGTGAGAACCCCGTGAGGCGAGATGTACCAGTTCTTTGTAGAGCGCTCTCGAAGAGTCGAGTTGTTTGGGAATGCAGCTCTAAGAGGGTGGTAAATTCCATCTAAAGCTAAATACAGGCGAGAGACCGATAGCGAACAAGTACAGTGATGGAAAGATGAAAAGAACTTTGAAAAGAGAGTGAAAAAGGACGTGAAATTGTTGAAAGGGAAGGGCATTTGATCAGACATGGTGTTTTGTGCCCCTCGCTCCTCGTGGGTGGGGGAATCTCGCAGCTCACTGGGCCAGCATCAGTTTTGGTGGCAGGAGAAAGCCTCGGGAATGTGACTCTTGCCTTTTTGGCGGGGGTGTTATAGCCCGAGGGGAATACTGCCAGCCGGGACTGAGGTATGCGACTCTCGTAGTCAAGGA

Strain CICY-RN-Sac2: *Saccharomyces cerevisiae* (519bp, Sac2_ brewing strain, F) D1/D2 LSU rDNA gene partial sequence. Strain obtained from research collaborators. Accession number of GenBank: MW267945

CGGCGAGTGAAGCGGCAAAAGCTCAAATTTGAAATCTGGTACCTTCGGTGCCCGAGTTGTAATTTGGAGAGGGCAACTTTGGGGCCGTTCCTTGTCTATGTTCCTTGGAACAGGACGTCATAGAGGGTGAGAATCCCGTGTGGCGAGGAGTGCGGTTCTTTGTAAAGTGCCTTCGAAGAGTCGAGTTGTTTGGGAATGCAGCTCTAAGTGGGTGGTAAATTCCATCTAAAGCTAAATATTGGCGAGAGACCGATAGCGAACAAGTACAGTGATGGAAAGATGAAAAGAACTTTGAAAAGAGAGTGAAAAAGTACGTGAAATTGTTGAAAGGGAAGGGCATTTGATCAGACATGGTGTTTTGTGCCCTCTGCTCCTTGTGGGTAGGGGAATCTCGCATTTCACTGGGCCAGCATCAGTTTTGGTGGCAGGATAAATCCATAGGAATGTAGCTTGCCTCGGTAAGTATTATAGCCTGTGGGAATACTGCCAGCTGGGACTGAGGACTGCGACGTAAGTCAA

Analytical conditions to measure gene expression of Vg, Po and GOx using Quantitative Real-Time PCR, in samples of Africanized *Apis mellifera* worker bees in Canche-Colli, et al. (MIQE Guidelines: Bustin et al. 2009. The MIQE Guidelines: Minimum Information for Publication of Quantitative Real-Time PCR Experiments. Clinical Chemistry 55:4: 611-622).

| **Sample/Template** | **details** |
| --- | --- |
| Source | Abdomen and head of *Apis mellífera* |
| Method of preservation | Liquid N2 |
| Storage time (if appropriate) | < 2 months |
| Handling | Frozen |
| Extraction method | TriZol |
| RNA: DNA-free | DNAse I protocol (DNA-free kit, Ambion, USA) |
| Concentration | Nanodrop (ND-1000 spectrophotometer) |
| RNA: integrity | Horizontal electrophoresis in agarose gel (1.5%) |
| Inhibition-free | RNAse inhibitor |
| **Assay optimisation/validation** | |
| Accession number | Vg (NM_001011578.1), proPO (AY242387.2), GOx (AB022907.1), RPS5 (XM_006570237.2) |
| Amplicon details | Vg (150 pb), proPO (130 pb), GOx (201 pb), RPS5 (115 pb) |
| Primer sequence | Vg (Fw: 5'- GTTGGAGAGCAACATGCAGA-3' and Rv: 3'-TCGATCCATTCCTTGATGGT-5'), proPO (Fw: 5'-GAACGGCTATGTAATCGTCTTGGA-3' and Rv: 3'- TACCGCTGGGTCGAAATGG-5'), GOx (Fw: 5'-GAGCGAGGTTTCGAATTGGA-3' and Rv: 3'-GTCGTTCCCCCGAGATTCTT-5'), RPS5 (Fw: 5'- AATTATTTGGTCGCTGGAATTG-3' and Rv: 3'-TAACGTCCAGCAGAATGTGGTA-5') |
| *Probe sequence** | Not used, our detection signal was SYBR Green Master Mix |
| *In silico* | BLAST and Primer Express™ Software vers. 3.0. Applied Biosystems™ |
| empirical | Primer concentration: 0.4 μM, Annealing temperature: Vg (58°C), proPO (58°C), Gox (59°C), RPS5 (56 to 62 °C) |
| Priming conditions | Target-specific |
| PCR efficiency | Dilution curve, Melting curve and Efficiency (above 95%) |
| Linear dynamic range | standard curve, serial dilutions with 5 points (300, 60, 12, 2.4, 0.48 Log Quantity) |
| Limits of detection | 50 ηg |
| Intra-assay variation | CVs (>10%) |
| **RT/PCR** | |
| Protocols | 10 μL of SYBR Green Master Mix (Applied Biosystems USA), 2 μL cDNA, 1μL of each gene-specific primer (0.4 μΜ final concentration) and 6 μL of ultre-pure water. |
| Reagents | SYBR Green Master Mix |
| Duplicate RT | DCq |
| NTC | Cq & melt curves |
| NAC | DCq beginning:end of qPCR |
| Positive control | Inter-run calibrators |
| **Data analysis** | |
| Specialist software | StepOne Software ver. 2.3 (Applied Biosystems, Foster City, CA, USA). |
| Statistical justification | Each sample had three internal replicates for accuracy |
| Transparent, validated normalisation | GeNorm summary / established method by Schmittgen and Livak, (2008). |

Treatment key for raw data in the repository (https://cicy.repositorioinstitucional.mx/jspui/handle/1003/1753)

| Treatment | Treatment key | Type of diet (yeast or essential oil added) |
| --- | --- | --- |
| Base diet | A | Control |
| S. bombicola | B | *Starmerella bombicola* |
| C. etchellsii | C | *Starmerella etchellsii* |
| S. bombicola 2 | D | *Starmerella bombicola* 2 |
| Z. mellis | E | *Zygosaccharomyces mellis* |
| S. cerevisiae | F | *Saccharomyces* *cerevisiae* |
| Carvacrol | G | EOs-chemotype-carvacrol |
| Thymol | H | EOs-chemotype-thymol |
| Sesquiterpenes | I | EOs-chemotype-sesquiterpenes |
